# 1551. Changes in Glomerular Filtration Rate after Switching from Tenofovir Disoproxil Fumarate to Tenofovir Alafenamide Fumarate for HIV Pre-exposure Prophylaxis

**DOI:** 10.1093/ofid/ofad500.1386

**Published:** 2023-11-27

**Authors:** Adovich Rivera, Matthew Mefford, Katherine J Pak, Rulin C Hechter

**Affiliations:** Kaiser Permanente Southern California, Pasadena, California; Kaiser Permanente Southern Caifornia, Pasadena, California; Kaiser Pemanente, Pasadena, California; Kaiser Permanente Southern California Department of Research and Evaluation, Los Angeles, California

## Abstract

**Background:**

In October 2019, tenofovir alafenamide fumarate (TAF) was approved for oral pre-exposure prophylaxis (PrEP) for preventing human immunodeficiency virus (HIV) infection. Compared with tenofovir disoproxil fumarate (TDF), TAF-containing regimens have demonstrated high efficacy and favorable changes in markers of renal function in randomized trials. While switching from TDF to TAF has been associated with improved renal function in people with HIV, this has not been assessed in the context of PrEP. Using electronic health records data from Kaiser Permanente Southern California (KPSC), we assessed changes in estimated glomerular filtration rate (eGFR) after switching from TDF to TAF.

**Methods:**

In this retrospective cohort study, KPSC members aged ≥18 years who filled a prescription of TDF for PrEP from October 2019-May 2022 were identified. Eligible individuals (Figure 1) who switched from TDF to TAF use during the study period were matched to 4 TDF users who did not switch at the switching date (index date) using time-varying propensity scores. A Bayesian linear mixed effects model was used to estimate differences in eGFR between switching and non-switching scenarios (i.e., “What would have happened to the eGFR if the switchers in the data did not switch?”) up to 18 months of follow-up. The model included spline terms for time and were adjusted for baseline eGFR and potential confounders. (Figure 2).

**Figure 1.**
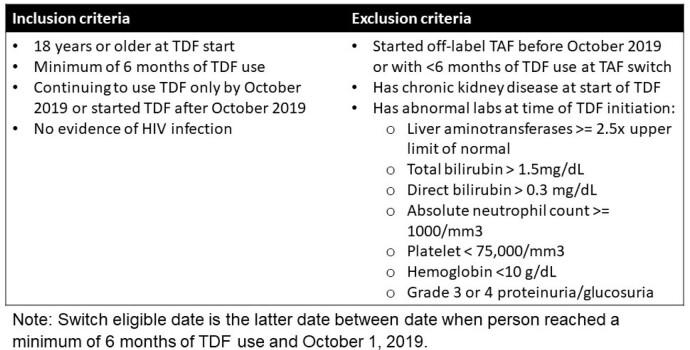
Eligibility criteria

**Figure 2.**
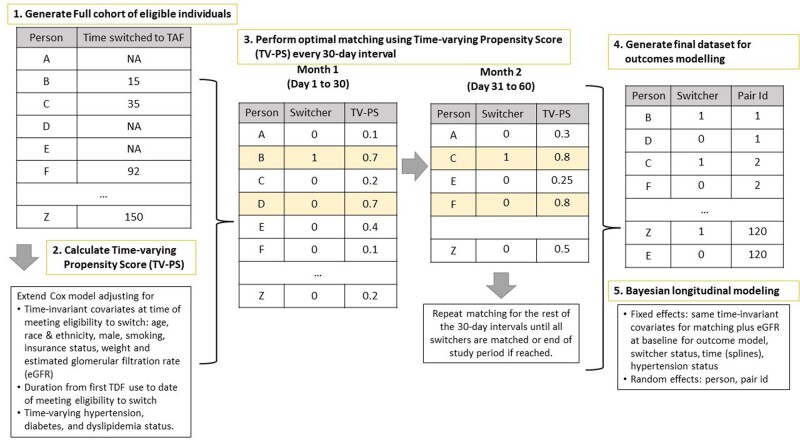
Steps in Creation and Analysis of the Matched Cohort

**Results:**

Among 5,250 eligible TDF users, 125 switched to TAF during follow-up. A total of 118 TDF to TAF switchers and 472 matched non-switchers were included in the analysis. Compared to non-switchers, switchers had similar baseline weight, lower baseline eGFR, but were more likely to be older age, White, on Medicare or Medicaid, and have hypertension. (Figure 3) Switching to TAF was associated with a higher eGFR compared to staying on TDF in 3-15 months post-switch, but 95% credible intervals cross the null. (Figure 4 and 5) The findings remained similar after excluding eGFR values after TAF switcher switched back to TDF or non-switcher started TAF. (Figure 5)

**Figure 3.**
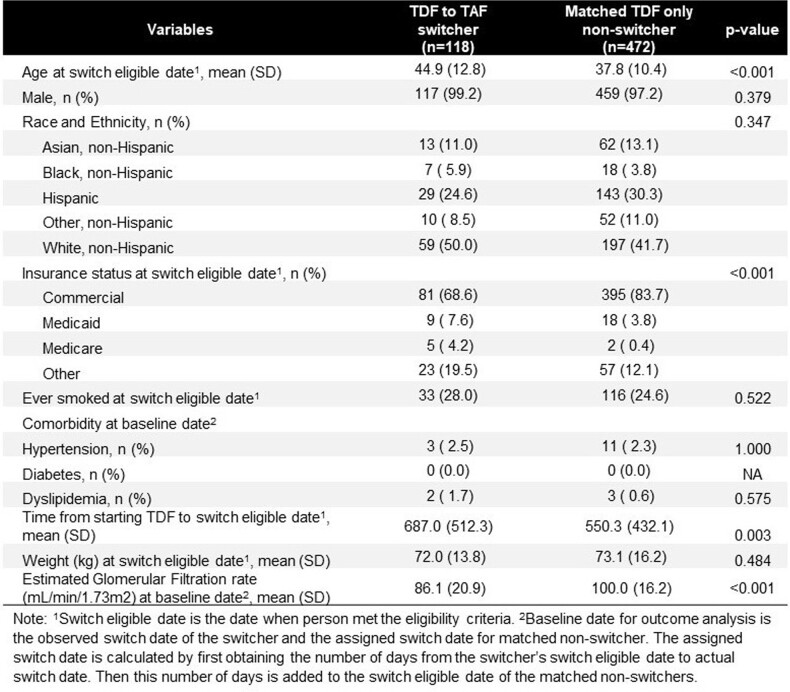
Comparison of Baseline Characteristics of TDF to TAF Switcher to Matched Non-Switchers.

**Figure 4.**
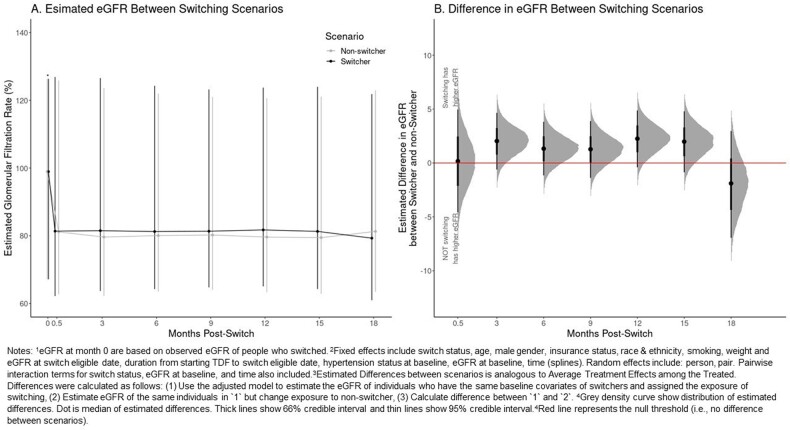
Distribution of Estimated Differences in Estimated Glomerular Filtration Rate between Two Scenarios at Selected Time Points: Switching from TDF to TAF compared to Staying on TDF (Not Switching)

**Figure 5.**
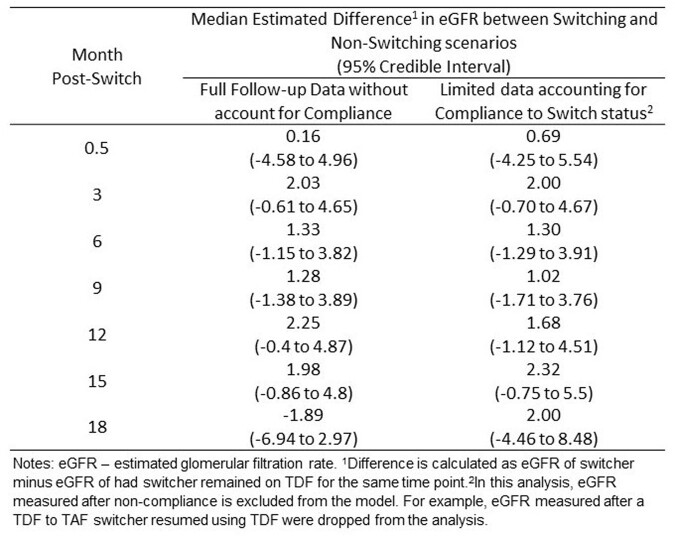
Median Estimated Difference in estimated Glomerular Filtration Rate using Full and Limited Data

**Conclusion:**

We found that switching from TDF to TAF may translate to a slightly better eGFR trajectory in the short term, but the difference might not be clinically important. Confirmation with larger datasets or randomized trials are important.

**Disclosures:**

**Matthew Mefford, PhD**, Merck & Co., Inc.: Grant/Research Support

